# Decadal change in seabird‐driven isotopes on islands with differing invasion histories

**DOI:** 10.1002/eap.70030

**Published:** 2025-06-02

**Authors:** Penelope P. Pascoe, Mitchell Bartlett, Justine Shaw, Rowan Trebilco, Christine K. Weldrick, Holly P. Jones

**Affiliations:** ^1^ University of Tasmania Institute for Marine and Antarctic Studies, Ecology and Biodiversity Hobart Tasmania Australia; ^2^ Independent Contractor Nelson New Zealand; ^3^ Securing Antarctica's Environmental Future Queensland University of Technology Brisbane Queensland Australia; ^4^ CSIRO Environment Hobart Tasmania Australia; ^5^ Australian Antarctic Program Partnership, Institute for Marine and Antarctic Studies University of Tasmania Hobart Tasmania Australia; ^6^ Department of Biological Sciences Northern Illinois University DeKalb Illinois USA; ^7^ Institute for the Study of the Environment, Sustainability, and Energy Northern Illinois University DeKalb Illinois USA

**Keywords:** chronosequence, ecosystem function, ecosystem recovery, eradication, rodents, seabird islands

## Abstract

Invasive mammal eradications are commonplace in island conservation. However, post‐eradication monitoring beyond the confirmation of target species removal is rarer. Seabirds are ecosystem engineers on islands and are negatively affected by invasive mammals. Following an invasive mammal eradication, the recovery of seabird populations can be necessary for wider ecosystem recovery. Seabirds fertilize islands with isotopically heavy nitrogen, which means that nitrogen stable isotope analysis (δ^15^N) could provide a useful means for assessing corresponding change in ecosystem function. We quantified decadal changes in δ^15^N on eight temperate New Zealand islands subject in pairs to distinct mammal invasion and seabird restoration histories: invaded, never‐invaded, invader‐eradicated, and undergoing active seabird restoration. First, we investigated long‐term changes in δ^15^N values on individual islands. Second, we used a space‐for‐time analysis to determine whether δ^15^N levels on islands from which invaders had been removed eventually recovered to values typical of never‐invaded islands. On each island, soil, plants (*Coprosma repens*, *Coprosma robusta*, and *Myrsine australis*), and spiders (Porrhothelidae) were sampled in 2006/2007 and 2022, allowing δ^15^N change on individual islands over 16 years to be assessed. Combined, the samples from invader‐eradicated islands provided a 7‐ to32‐year post‐eradication dataset. Change in δ^15^N was only detected on one island across the study period, following the unexpected recolonization of seabirds to an invaded island. Invader‐eradicated islands generally had higher δ^15^N values than invaded islands; however, they were still lower than never‐invaded islands, and there was no trend in δ^15^N with time since eradication. This, and the measurable increase in δ^15^N following seabird recolonization on one island, may suggest that δ^15^N change occurs rapidly following invader eradication but then slows, with δ^15^N values staying relatively constant in the time period studied here. Isotope and seabird population studies need to be coupled to ascertain whether plateauing in δ^15^N reflects a slowing of seabird population growth and subsequent basal nutrient input or whether the baseline nutrients are entering the ecosystem but then not propagating up the food web.

## INTRODUCTION

Islands play a key role in the global effort to conserve biodiversity (Holmes et al., 2019). They are home to a disproportionate number of endemic and endangered taxa and provide refuges for numerous species from the anthropogenic disturbances found on mainlands (Spatz, Zilliacus, et al., 2017; Tershy et al., 2015). Island taxa are also at disparate risk of extinction, with 61% of all species listed as extinct by the International Union for Conservation of Nature (IUCN) endemic to islands (Tershy et al., 2015). Invasive mammalian predators are one of the biggest threats to native taxa on islands (Doherty et al., 2016), with invasive rodents being one of the most pervasive groups (Howald et al., 2007).

Invasive mammal eradications are now recognized as an important tool for conserving island ecosystems (Jones et al., 2016; Spatz et al., 2022). Over 1500 eradications have been successfully completed across more than 960 islands to date, defined as the total removal of a target species (DIISE, 2018). Monitoring beyond confirming the successful removal of invasive species remains rare, leaving a lack of understanding of ecosystem responses to invasive mammal removal (Jones et al., 2016). Post‐eradication monitoring is time‐ and resource‐intensive (Bird et al., 2019) and generally only focused on a small number of species, which may not represent the rest of the ecosystem (Levin, 1998). Defining a post‐eradication “end‐point” is also challenging with pre‐invasion baseline data rarely available (Bellingham et al., 2010; Simberloff, 1990). However, understanding the response of island ecosystems to eradications is important. It provides information for making future evidence‐based conservation decisions such as where to allocate limited conservation budgets and when follow‐up restoration efforts are required (Buxton et al., 2016; Spatz et al., 2022; Sutherland et al., 2004; Wortley et al., 2013).

Seabirds profoundly affect the island ecosystems on which they breed such that “seabird islands” are recognized as unique and important ecosystems globally (Mulder, Anderson, et al., 2011). By foraging at sea and then returning to islands to breed, seabirds transport nutrients from the marine environment to terrestrial island ecosystems through their guano and carcasses (Gillham, 1956a; Smith et al., 2011). They also cause physical changes through burrowing, nest building, and trampling (Gillham, 1956b; Smith et al., 2011). These physical and chemical alterations have the potential to increase primary productivity (Polis et al., 1997; Stapp et al., 1999), alter soil and vegetation properties (Ellis et al., 2011; Fukami et al., 2006; Grant et al., 2022; Kolb et al., 2010; Mulder, Jones, et al., 2011), and shape consumer and predator communities (Kolb et al., 2011; Polis & Hurd, 1995; Sanchez‐Pinero & Polis, 2000; Towns et al., 2009).

Rodents threaten seabirds via predation on eggs and chicks, reducing breeding success, and pose numerous direct and indirect threats to the rest of an island's ecosystem (Towns et al., 2006). The key role of seabirds in island ecosystems means that their suppression, and the resulting loss of seabird‐driven disturbance and nutrient subsidies, leads to ecosystem‐wide changes in the presence of invasive rodents (Ellis et al., 2011; Fukami et al., 2006; Wardle et al., 2009). The ecosystem‐engineering role that seabirds play on islands means that their numerical population responses could be a proxy for an island's ecosystem‐wide response to an eradication. However, monitoring even large species such as seabirds is challenging, with population estimates often having high uncertainty (Bird et al., 2021). While seabird populations often increase following invasive mammal eradications (de Brooke et al., 2017), this is not always the case (Buxton et al., 2014; Gaze, 2000). Where seabirds have been extirpated or greatly reduced on an island, their recolonization can be a delayed and slow process due to their strong breeding site philopatry (Bird et al., 2022; Buxton et al., 2014). In some instances, active seabird restoration may be required, such as chick translocations or acoustic attraction of breeding birds to a site (Buxton et al., 2016). Seabirds are long‐lived and slow‐breeding (Warham, 1990), meaning that decades of repeated monitoring are required to obtain population trends. Additionally, due to complex interactions among components of the island ecosystem, the return of the ecosystem to its pre‐invaded state can be unpredictable even if seabird numbers rebound. Change may also manifest in more subtle measures than abundance, such as shifts in behavior or interaction patterns, which can alter ecological processes (Zavaleta et al., 2001).

**FIGURE 1:**
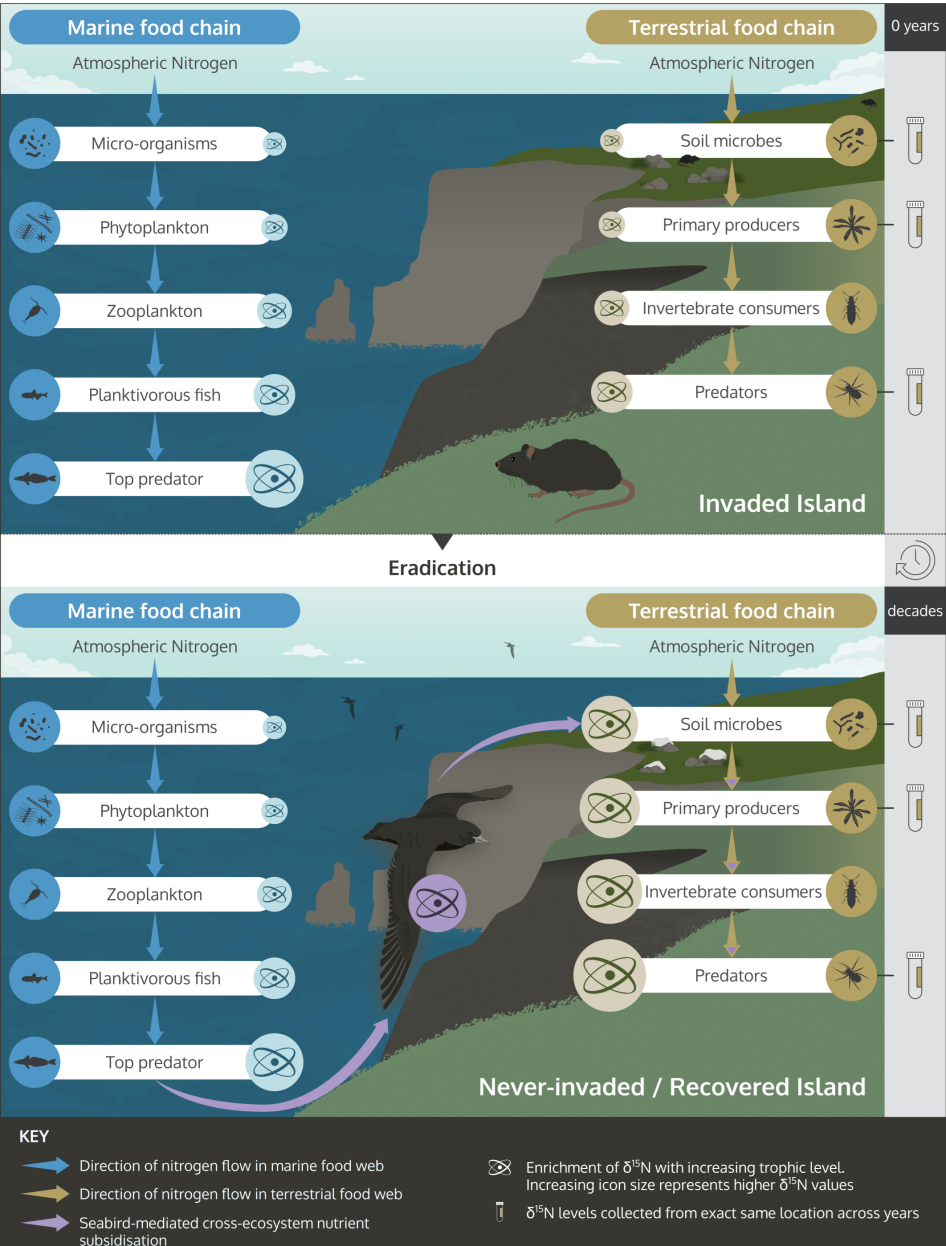
Conceptual framework for the use of nitrogen stable isotope analysis as a tool for monitoring. Graphic design: Stacey McCormack (Visual Knowledge).

One means for investigating ecosystem change following invasive mammal eradications is to track seabird influence through island food webs via the use of nitrogen stable isotope analysis (SIA_N_) (Jones, 2010a; Nigro et al., 2017) (Figure 1). Stable isotopes are naturally occurring variants of an element, differing only in their number of neutrons (Fry, 2006; Peterson & Fry, 1987). Analysis of the relative abundance of the two commonly occurring isotopes of nitrogen, ^15^N and ^14^N, is commonly used in trophic studies, as the lighter nitrogen isotope ^14^N is preferentially lost in excreta at each trophic level, and consequentially, the ratio of ^15^N to the lighter ^14^N (expressed as δ^15^N) increases with trophic level (Peterson & Fry, 1987). Seabirds forage near the top of the marine food web, so they are consuming prey enriched in ^15^N. On islands with nesting seabirds, the terrestrial ecosystem is “fertilized” with this enriched ^15^N when seabirds deposit guano and through carcass, shell, and feather decomposition (Smith et al., 2011) (Figure 1). This subsidy makes the δ^15^N values of terrestrial ecosystem components on islands with seabirds higher than those on islands without seabirds (Croll et al., 2005; Fukami et al., 2006; Jones, 2010a; Maron et al., 2006; Mulder et al., 2009). On islands where invasive species have been eradicated, δ^15^N values have been found to be higher than those on islands where invasive species are still present but still lower than those on islands that have never had invasive species (Jones, 2010a, 2010b). Consequentially, SIA_N_ may provide a useful means for assessing ecosystem recovery on invader‐eradicated seabird islands. Additionally, sample collection for stable isotope analysis is low‐impact and relatively quick and simple, compared to the extensive time required to adequately survey seabirds, particularly burrowing species. Nitrogen stable isotope analysis also provides inferences about the broader ecosystem state which may be challenging to monitor directly or may reveal important cryptic ecosystem components or interactions between species.

Little is known about how δ^15^N values change on individual islands, particularly over decadal time frames. Existing longitudinal isotope studies on individual islands have only spanned short time frames (e.g., months to 6 years, Caut et al., 2012; Nigro et al., 2017) or focused on seasonal changes in isotopic values or the effects of seabird breeding cycles (Gaiotto et al., 2022; Pascoe et al., 2022) or seasonal weather patterns (Gaiotto et al., 2022; Stapp et al., 1999) on δ^15^N levels. Understanding how δ^15^N values change on individual islands over longer time frames and how this differs between islands of differing conservation or restoration status is critical to investigate whether SIA_N_ is to be developed further as a post‐eradication ecosystem function assessment tool.

However, investigating long‐term change of any type on remote and logistically hard‐to‐access islands is a challenge. It is generally not possible to visit and sample an island repeatedly for decades following an eradication to assess ecosystem change due to logistical challenges and resource limitations. One method for addressing this is to use space‐for‐time substitution (also known as chronosequence analysis). This involves sampling different islands representing varying times since eradication of invaders to make inferences about how post‐eradication ecosystems may behave over a longer time frame. A potential endpoint of island restoration or recovery may be established using as references nearby and environmentally similar islands that have never been invaded (Jones, 2010a; Towns et al., 2016; White & Walker, 1997). Using such an analysis across 15 islands in northern New Zealand, an original study by Jones (2010a) predicted that it would take 39, 28, and 32 years post rodent eradication for soil, plant (*Coprosma repens*), and spider (family Porrhothelidae) δ^15^N values, respectively, to reach levels similar to those found on never‐invaded islands. Scalable eradication technologies were not developed until the 1990s (Towns & Broome, 2003), so until recently there have not been any studies with long enough time series to test whether these predicted “recovery” times are correct.

In this study, we investigate long‐term δ^15^N change on islands in two ways. First, we resample δ^15^N of soil, plants, and spiders from eight islands in Cook Strait, New Zealand, originally sampled by Jones (2010b) 16 years earlier. This allowed us to assess, for the first time, decadal scale changes in δ^15^N on islands never invaded and invaded by mammals and on islands with invasive mammals eradicated. We then test the recovery time predictions made on islands in northern New Zealand by Jones (2010a) by comparing δ^15^N values for islands from which invaders have been eradicated for 7–32 years to those of invaded and uninvaded islands.

We hypothesize that on never‐invaded islands, if seabird populations are at their carrying capacity and the ecosystem is stable, δ^15^N values would be high, but there would be little change in δ^15^N values over time. On islands invaded by rodents, suppressing seabird populations and the input of marine nutrients and disrupting ecosystem linkages, δ^15^N values would be low but may also remain stable over time. With invader‐eradicated islands known to have elevated δ^15^N values compared to invaded islands, we hypothesize that these would continue to increase over time, and for this to be even more pronounced on islands where seabird recovery is being facilitated by active seabird restoration efforts.

Specifically, we aimed to answer the following questions:

1. Do δ^15^N values change on individual islands over the 16‐year study period, and does island restoration treatment (invaded, never‐invaded, invader‐eradicated, or actively seabird restored) influence this change?
2. Following an eradication, do ecosystem components attain δ^15^N values similar to never‐invaded islands within the previously predicted (see Jones, 2010a) 28–39 years?

## METHODS

### Study islands

**FIGURE 2:**
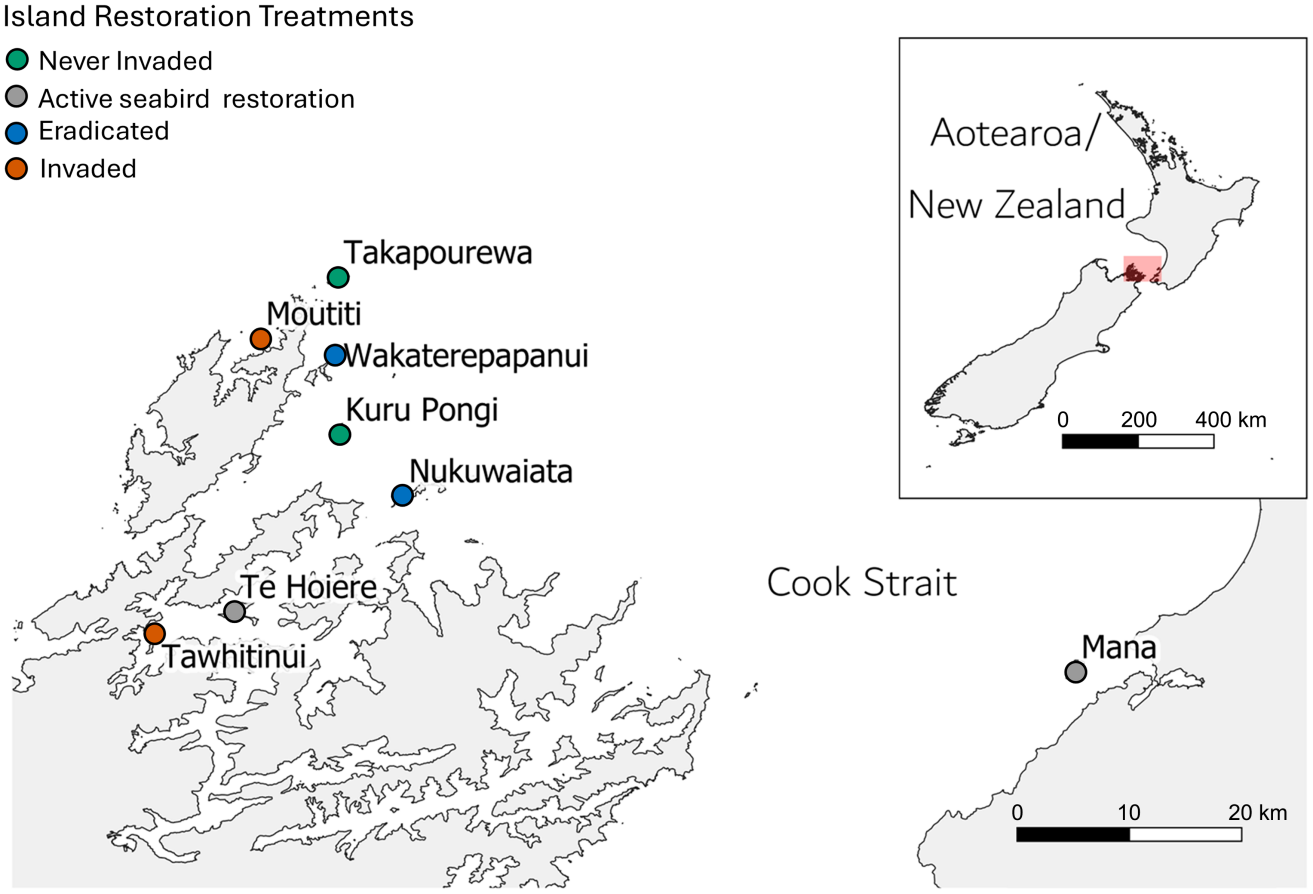
Study island locations and respective restoration treatments in Cook Strait, Aotearoa/New Zealand. Each island was sampled in 2006/2007 and 2022. Takapourewa and Kuru Pongi have never been invaded by rodents; Moutiti and Tawhitinui are still invaded by rodents. Te Hoiere, Mana, Wakaterepapanui, and Nukuwaiata have all undergone rodent eradications (*Mus musculus*—2014, *Mu. musculus*—1990, *Rattus exulans* and *Rattus norvegicus*—1999, and *R. exulans*—1994, respectively). Mana and Te Hoiere have also had active seabird restoration projects, commencing in 1993 and 1991, respectively.

We selected eight geographically proximate and environmentally similar islands in Cook Strait, New Zealand, to investigate long‐term changes in nitrogen stable isotope levels across islands with different restoration treatments: never‐invaded islands—Takapourewa/Stephens and Kuru Pongi/Middle Trio, invaded islands—Moutiti/Victory and Tawhitinui, invader‐eradicated islands—Wakaterepapanui and Nukuwaiata, and islands with active seabird restoration—Te Hoiere/Maud and Mana (Figure 2). Each island was originally sampled for a stable isotope study in 2006/2007 by Jones (2010b), and its restoration treatment status was defined specifically in relation to its rodent invasion history. We revisited each island in 2022 for this study.

Takapourewa and Kuru Pongi have never been invaded by rodents, providing positive controls for what the other islands may look like today without a history of rodent invasion and a proxy for ecosystem function “recovery” for the invader‐eradicated islands. Both islands have high densities of breeding seabirds such as tītī wainui/fairy prion (*Pachyptila turtur*), tītī/sooty shearwaters (*Ardenna grisea*), northern diving petrels (*Pelecanoides urinatrix urinatrix*), and pakahā/fluttering shearwaters (*Puffinus gavia*), with the largest colony of fairy prion potentially in the world, found on Takapourewa (Jamieson et al., 2016). On Takapourewa, the population of fairy prions was thought to have increased from 1975 to 1998, likely a response to habitat improvements following the end of farming and the planting of new forests, although counts were inaccurate (Jamieson et al., 2016). It is now thought that the island may be at carrying capacity, and it is unlikely that there has been significant population change on either Takapourewa or Kuru Pongi in recent decades (G. Taylor, personal communication). Moutiti and Tawhitinui are still invaded by rodents (*Rattus* spp.), and in 2006/2007, seabird populations were undetectable on these islands, providing negative controls from which to assess change since eradication. Te Hoiere, Mana, Wakaterepapanui, and Nukuwaiata have all undergone rodent eradications (*Mus musculus—*2014, *Mu. musculus—*1990, *Rattus exulans* and *Rattus norvegicus—*1999, and *R. exulans—*1994, respectively) (Table 1); however, the presence of rodents on Te Hoiere only lasted from October 2013 to 2014 when they were eradicated (Pichlmueller et al., 2020). In 2006/2007, Nukuwaiata had small remnant colonies of sooty and fluttering shearwaters, but Wakaterepapanui had no colonial seabirds present (Jones, 2010b). Mana and Te Hoiere have also had active seabird restoration projects, commencing in 1993 and 1991, respectively (Bell et al., 2005) (Table 1). On Te Hoiere, 334 fluttering shearwaters chicks were transferred from 1991 to 1996. By 2003/2004, there were only 15 breeding pairs (Bell et al., 2005). The restoration colony on Te Hoiere has not been actively monitored in recent years. The island also does not have any natural seabird colonies, for unknown reasons (Jones, 2010b). Active restoration on Mana commenced in 1993 with acoustic attraction of adult kuaka/common diving petrel (*Pelecanoides urinatrix*), followed by the translocation of 239 chicks from 1997 to 1999 (Miskelly & Taylor, 2004). From 2002 to 2004, 240 fairy prion chicks were also translocated (Miskelly & Gummer, 2013) and supplemented with an additional 200 chicks from 2015 to 2016 (Gummer et al., 2016). From 2006 to 2008, 225 fluttering shearwaters were also translocated to Mana (Gummer & Adams, 2010). As of the 2021/2022 breeding season, there were 14, 9, and 40 fledging chicks of each species, respectively, on the island (Miskelly, 2023). Mana Island also has natural colonies of sooty shearwaters, with around 100 chicks banded in 2019 (Miskelly, 2023) (Table 1).

**TABLE 1:** Restoration treatment and dominant seabird species for the eight Cook Strait study islands.

Island (Māori/English name)	Size (ha)	Restoration treatment (rodent status)^1^	Invasion/restoration treatment details	Dominant seabird species	Seabird density 2006/2007	Seabird density 2022: Burrow entrances/m^2^ (no. colony plots)
Takapourewa/Stephens	150	Never‐invaded	~1895 to ~1925: *Felis catus* ^2^	Fairy prions, sooty shearwaters, northern diving petrels, fluttering shearwaters^1^	High	High
Kuru Pongi/Middle Trio	13	Never‐invaded		Fairy prions, sooty shearwaters, northern diving petrels, fluttering shearwaters^1^	High	High
Moutiti/Victory	16	Invaded	*Rattus* spp.	Blue penguin, shearwater spp.^a^ ^,11^	Undetectable	Low
Tawhitinui	22	Invaded	*Rattus* spp.		Undetectable	Undetectable
Wakaterepapanui	74	Eradicated	1999: *Rattus exulans* and *R. norvegicus* eradicated^3^		Undetectable	Undetectable
Nukuwaiata	249	Eradicated	1994: *Rattus exulans* eradicated^3^	Sooty and fluttering shearwaters^11^	Low	Low
Te Hoiere/Maud	309	Seabird restoration	1991–1996: fluttering shearwaters,^4^ 2013: house mouse invaded,^5^ 2014: house mouse eradicated^5^	Fluttering shearwaters^4—^restoration colony only	Undetectable outside of restoration colony	High in restoration plots, undetectable outside of restoration colony
Mana	217	Seabird restoration	1990: house mouse eradicated,^6^ since 1997–2016: common diving petrel,^5,10^ fairy prions,^7,8,10^ and fluttering shearwaters^9,10^ translocated	Sooty shearwaters (natural colony), common diving petrel,^5^ fairy prions,^7,8^ and fluttering shearwaters^9^ (restoration colony)	Low	Very high in restoration plots, undetectable outside of restoration colony

*Note*: “Restoration Treatment” was initially categorized by Jones (2010b) and referred to rodent invasion history. “Restoration Treatment Details” provide additional information on invasions, eradications, and seabird translocations. (1) Jones (2010b), (2) Brown (2000), (3) Howald et al. (2007), (4) Bell et al. (2005), (5) Miskelly and Taylor (2004), (6) DIISE (2018), (7) Miskelly and Gummer (2013), (8) Gummer et al. (2016), (9) Gummer and Adams (2010), (10) Miskelly, 2023, (11) Authors' observations.

^a^ Absent in 2006/2007.

Seabird population monitoring beyond what is described above is relatively sparse across most of the islands over the study period, and pre‐invasion baseline data are not available due to the long history of rodent presence on the islands. In the absence of available comparable seabird population density data, each island was assigned as having high, low, or undetectable burrowing seabird numbers by Jones (2010b) during the initial 2006/2007 stable isotope sampling visit (Table 1). In 2022, we reassessed seabird numbers on each island by counting the number of burrow entrances in a plot of 3 m radius around each within‐colony sampling point (2–4 plots per island) and calculating the mean ± 1 SD of burrow entrances per m^2^ for all within‐colony plots for each island. Burrow occupancy was not assessed. While not quantitatively comparable to the 2006/2007 colony assessments, this allowed us to detect any dramatic intra‐island changes between the two sampling events. For ease of comparison with the original burrow density estimates, these were then also converted to low‐, medium‐, and high‐density scores based on their relativity to each other (Table 1).

### Sample collection and stable isotope analysis

In the original study by Jones (2010b), soil, plant (taupata—*Coprosma repens*, karamu—*Coprosma robusta*, and māpou—*Myrsine australis*), and spider (family Porrhothelidae) samples were collected from inside and outside seabird colonies (no burrows found within a 3‐m radius of the sampling location) at between 9 and 47 sampling locations on each island in October to December 2006 and/or 2007 to investigate the effects of invasive rodent eradication and seabird restoration on ecosystem function recovery. Methods are outlined in (Jones 2010b). In brief, at each sampling location, soil and as many of the three plant species as were present were collected. Plants were sampled by picking three new growth leaves from separate individuals of the same species or from the same plant if multiple plants were not present. Soil was sampled by scraping back the litter layer and collecting approximately 100 g using a small hand trowel down to 10 cm depth. Up to three spiders were collected from inside and three from outside seabird colonies opportunistically on each island (because they were not always present in each sampling locale) by digging up their tunnels.

Of these initial sampling locations selected by Jones (2010b), we resampled up to six of them from February to April 2022, selecting three from inside a seabird colony and three outside a seabird colony, where possible on each island as the effects of seabirds can be quite localized (on invaded islands with no seabirds all sampling occurred outside colonies) (Pascoe et al., 2022). All sampling was conducted during the seabird breeding season, and our previous work on seasonal variability in δ^15^N on temperate islands in New Zealand and Tasmania, Australia, suggests that little temporal variability in δ^15^N should be expected due to the different sampling months (Pascoe et al., 2021, 2022). On each island with a seabird colony, we also collected three seabird guano samples to allow the baseline δ^15^N entering each island to be measured.

Spider samples were stored in 70% ethanol for transportation off the island. All samples were then washed in distilled water and oven dried at 60°C for 48 h to prevent decomposition and in preparation for stable isotope analysis. Dried soil samples were passed through a 0.5‐mm sieve, and then, all samples were hand ground to a fine powder using a mortar and pestle.

Stable isotope analysis was conducted using an Elemental Combustion System (ECS 4010, Costech Instruments) coupled to the Delta Plus Advantage IRMS (Thermo Fisher Scientific) mass spectrometer at Northern Illinois University using USGS‐25 and IAEA‐N1 international standards. Stable isotope abundances were reported in delta (δ) values, which are the deviation of a sample's ^15^N:^14^N ratio from that of atmospheric nitrogen in parts per mil (‰), and is calculated from the following equation:

$$\delta X\left(‰\right)=\left[\left(R_{\text{sample}}/R_{\text{standard}}-1\right)\times 1000\right],$$

where *X* = ^15^N and *R* = the ratio of ^15^N:^14^N. Precision expressed as 1 SD combined uncertainty was between 0.18‰ and 0.25‰ for different in‐house standards calibrated with USGS‐25 and IAEA‐N1.

### Statistical analysis

All analyses were conducted in R studio (RStudio Team, 2024) using R version 4.0.2 (R Core Team, 2024). Code and raw data used are available at https://doi.org/10.25959/VDN6-R115. Means are expressed as ±1 SD. Model assumptions were assessed using QQ plots and residuals versus linear predictor plots.

#### Long‐term δ^15^N change on individual islands

To assess changes in δ^15^N over the study period on each island and to investigate whether island restoration treatment (invaded, never‐invaded, invader‐eradicated, or seabird restoration) influenced the magnitude of these changes, we compared soil, plant, and spider samples collected from the exact same locations (with ±3 m GPS error) in 2006/2007 and 2022 (*n* = up to 6 replicated sampling locations per island), hereafter referred to as the “initial” and “new” sampling events, respectively. We considered exact spatial and sample type (down to species for plant and family for spiders) replication necessary as we previously identified these factors as strong contributors to variation in δ^15^N values (Pascoe et al., 2022), which would confound the change in δ^15^N in response to the time between the sampling events. Where samples of the same type were collected from the same location in both 2006 and 2007, we took the average to generate a single initial isotope value as any differences between the two years would likely be due to interannual variation rather than a response to restoration treatment. As spider samples were collected opportunistically, spatial replication was not possible. As sampling inside or outside seabird colonies is known to have a significant effect on δ^15^N levels across ecosystem components (Jones, 2010b; Pascoe et al., 2021, 2022), we took the average of all spiders collected inside, and all spiders collected outside seabird colonies at the initial and new sampling events were to be used as replicate samples. This gave us up to six initial and new δ^15^N values for soil and each plant species and up to two δ^15^N values for spiders for each of the eight islands (for exact numbers of each sample type collected at each island, see Appendix S1: Table S1). While having more than just two temporal sampling points would be preferable for ascertaining trends, the logistical challenges of conducting long‐term studies on remote islands made this impossible at this stage.

We investigated whether δ^15^N values changed across the islands between the initial and new sampling events by fitting a linear mixed‐effects model using the “lmer” function from R package “lme4” (Bates et al., 2015). The interaction between island and sampling event was examined with sampling unit (the replicated sample) as a random effect to account for variation between sampling locations and sample types and sample δ^15^N as the response variable (Appendix S1: Section S1.1). To investigate whether the magnitude of the change in δ^15^N over the study period was influenced by island restoration treatment (invaded, never‐invaded, invader‐eradicated, or seabird restoration), whether different sample types (*C. repens*, *C. robusta*, *My. australis*, soil, or spider) responded more or less rapidly, or whether colony status (inside or outside) influenced the magnitude of the change, we then fit a separate linear model (function “lm” from R package “stats,” R Core Team, 2023) with sample type, restoration treatment, and colony status as the fixed effects and the magnitude of change in δ^15^N values for a replicated sample (δ^15^N_new_ − δ^15^N_initial_) as the response variable (Appendix S1: Section S1.2). The inclusion of island as an additional additive fixed effect was investigated but did not improve the model (dAIC = 5.10).

#### Multi‐decadal δ^15^N trends across invader‐eradicated islands using space‐for‐time substitution

For δ^15^N values from different islands to be comparable in a space‐for‐time substitution analysis, we first needed to ensure that the basal δ^15^N value entering each islands' terrestrial ecosystem from seabirds was comparable. Seabird guano samples were available for stable isotope analysis from all never‐invaded, invader‐eradicated, and seabird‐restoration islands, aside from Mana where guano samples deteriorated prior to analysis and could therefore not be reliably used. We used ANOVA to compare the guano δ^15^N values between islands (function “lm” from R package “stats,” R Core Team, 2023).

To investigate long‐term δ^15^N trends across the invader‐eradicated islands, samples from 2006 and 2007 were used as separate data points to accurately represent the years since eradication and compared to the 2022 sampling event, yielding up to three sampling events per island. For comparability with the original predictive study from northern New Zealand (Jones, 2010a), and because we (1) had fewer inside colony samples (Appendix S1: Table S1), and (2) the effect of burrowing seabirds on these and other temperate islands is quite localized (Jones, 2010b; Pascoe et al., 2022), we only used samples from the invader‐eradicated islands collected outside seabird colonies to ensure that the samples reflected the island‐wide dynamics rather than the localized seabird effect. Insufficient samples were available to build trends for *C. robusta* or *My. australis* plants, so these were not analyzed.

We plotted and fitted separate linear models (function “lm” from R package “stats,” R Core Team, 2023) to the δ^15^N value for the remaining sample types (soil, spider, and *C. repens*) collected on each invader‐eradicated island against the island's time since eradication at the time of sample collection (Appendix S1: Section S1.3). We then compared these trends with the average δ^15^N level for the same sample type collected from the never‐invaded islands as a proxy for the level the samples may reach when the ecosystem has “recovered” and the invaded islands as a starting point. For the never‐invaded islands, only inside‐colony locations were sampled as the only colony‐free areas were generally close to the coast. The latter areas may be influenced by other marine subsidies such as beach wrack, as this organic matter washing onto beaches is known to provide a source of nutrients to coastal species such as spiders (Anderson & Polis, 1998). Together, the samples collected in 2006/2007 and 2022 gave us an invader‐eradicated island space‐for‐time substitution dataset of 7–32 years.

## RESULTS

### Long‐term δ^15^N change on individual islands

**FIGURE 3:**
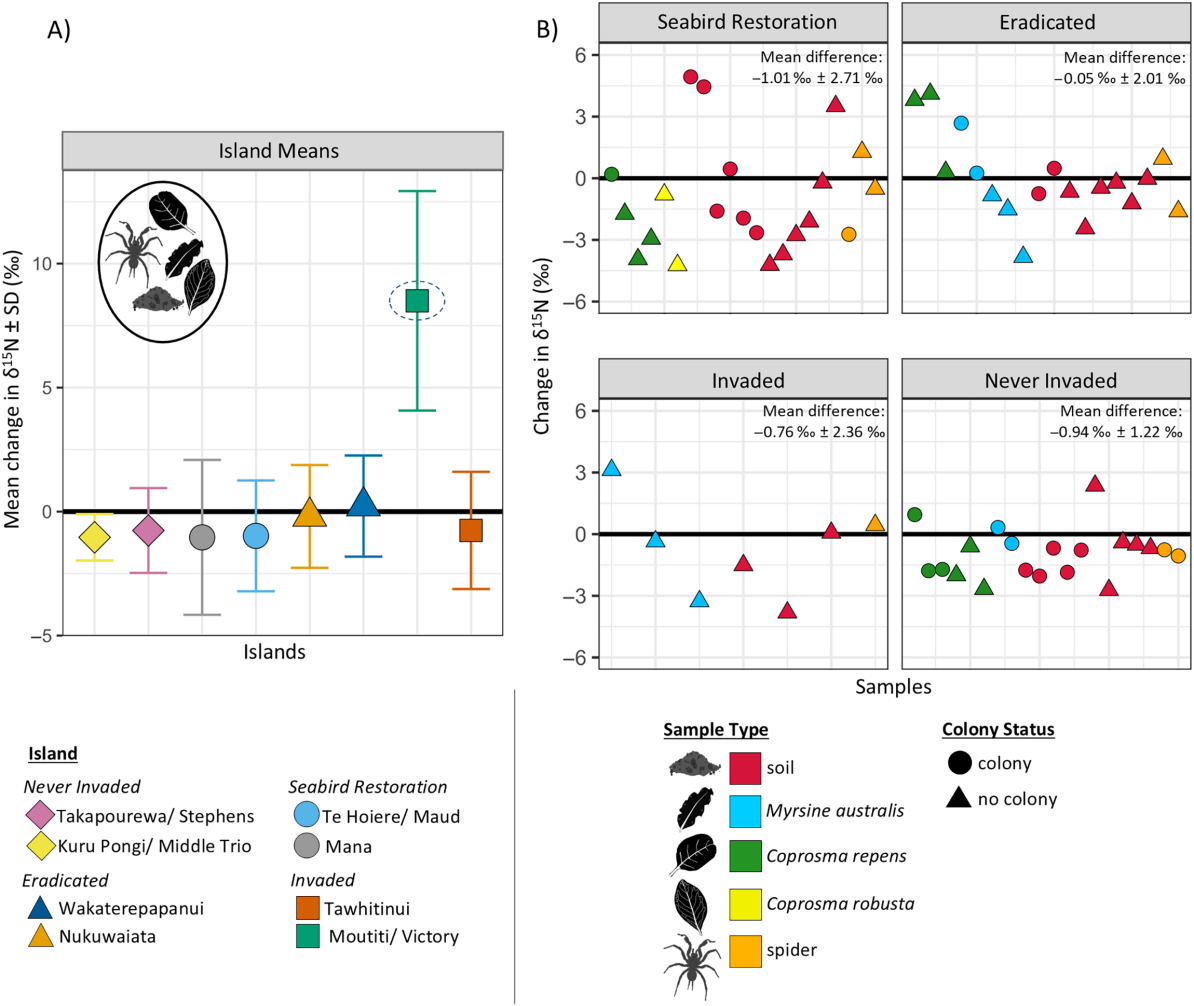
(A) Mean change in nitrogen stable isotope (δ^15^N) values between 2006/2007 and 2022 sampling events (±1 SD) for replicate soil, plant (*Coprosma repens*, *Coprosma robusta*, *Myrsine australis*), and spider samples combined for each study island. The broken circle highlights Moutiti/Victory Island which, following seabird recolonization, was no longer considered a representative invaded control island and thus omitted from panel (B). (B) Change in replicate soil, plant, and spider samples displayed separately and broken up by island restoration treatment (facets), sample type (colors), and seabird colony status at the sampling location (shapes). For both panels, islands/samples falling below *Y* = 0 (bold line) have decreased in δ^15^N between the 2006/2007 and 2022 sampling events. Icon illustrations: Penelope Pascoe.

There was no significant change in average δ^15^N between the initial and new sampling events for any of the islands, except for Moutiti (*p* < 0.001) where average δ^15^N increased by 8.5‰ ± 4.4‰ over the 16‐year study period (Figure 3A, Table 2). When the islands were visited in 2006/2007 as part of the original study by Jones (2010b), Moutiti Island was selected as an invaded control island based on the presence of rodents and an undetectable seabird presence. When revisited in 2022, active and established seabird colonies of kororā, blue penguin (*Eudyptula minor*), and potentially shearwaters (unconfirmed) were observed on Moutiti (Table 1). The notable increase in δ^15^N values across sample types on this island likely indicates that SIA_N_ was able to detect this dramatic ecological change on the island.

**TABLE 2:** Summary of model output for the effect of sampling event (2006/2007 or 2022) on δ^15^N values for each island.

Fixed effect	Predicted δ^15^N (95% CI) for sampling events		*t* value	*p*
Island × sampling event	Initial	New		
Kuru Pongi/Middle Trio	16.40 (14.35, 18.44)	15.36 (13.31, 17.41)	−1.39	0.17
Mana	10.40 (8.27, 12.53)	9.36 (7.23, 11.49)	0.00	1.00
Te Hoiere/Maud	6.24 (3.78, 8.69)	5.26 (2.80, 7.72)	0.05	0.96
Moutiti/Victory	6.43 (4.39, 8.48)	14.94 (12.89, 16.98)	9.06	**<0.001**
Nukuwaiata	11.85 (9.72, 13.98)	11.66 (9.53, 13.79)	0.78	0.43
Takapourewa/Stephens	16.39 (13.60, 19.17)	15.62 (12.84, 18.41)	0.22	0.83
Tawhitinui	−1.11 (−3.89, 1.68)	−1.86 (−4.65, 0.92)	0.22	0.83
Wakaterepapanui	5.75 (2.74, 8.76)	5.97 (2.96, 8.98)	0.95	0.34

*Note*: Sampling unit (replicated sample collected in the same location on each island in each sampling event) was included as a random effect, with the interaction between island and sampling event as the fixed effect. The predicted initial and new δ^15^N values with 95% CIs are provided. Random effect = Sampling unit; marginal *R* ^2^ = 0.67; conditional *R* ^2^ = 0.91.

With the colonization of seabirds and the subsequent increase in δ^15^N recorded on Moutiti Island across sample types (Figure 3A), we no longer considered it to be representative of what an invaded island would look like isotopically and therefore no longer a suitable invaded control island. Hence, it was removed from analysis investigating the effect of restoration treatment. As it is unknown when seabirds returned to the island, it was also not possible to investigate isotopic change over time (in a similar way to the invader‐eradicated islands). For replicated samples across the other islands, there was no effect of sample type (ANOVA, *F*_4,57_ = 0.44, *p* = 0.78), colony status (ANOVA, *F*_1,57_ = 0.87, *p* = 0.36), or island restoration treatment (ANOVA, *F*_3,57_ = 0.99, *p* = 0.40) on the change in δ^15^N values between the sampling events, further highlighted by a negative adjusted *R* ^2^ value, indicating that none of the predictor variables had strong predictive value (Figure 3B, Table 3). Never‐invaded islands showed the smallest variance in δ^15^N (±1.2‰ SD), and actively restored islands had the largest variance (±2.7‰ SD) (Figure 3B). Mean δ^15^N values for each sample type from each island are provided in Appendix S1: Table S1.

**TABLE 3:** Summary of model output for the effect of sample type (*Coprosma repens*, *Coprosma robusta*, *Myrsine australis*, soil, or spider), colony status (inside or outside), and island restoration treatment (invaded, never invaded, invader eradicated, or seabird restoration) on the change in δ^15^N value between 2006/2007 and 2022.

Response	Fixed effects	Intercept			
		Estimate	SE	*t* value	*p*
New δ^15^N ‐ Initial δ^15^N values	(Intercept)	−0.11	0.86	−0.13	0.90
	Sample type: *Coprosma robusta*	−1.50	1.70	−0.89	0.38
	Sample type: *Myrsine australis*	−0.36	1.00	−0.36	0.72
	Sample type: soil	−0.34	0.72	−0.47	0.64
	Sample type: spider	−0.09	0.98	−0.09	0.93
	Colony status: out	−0.89	0.62	−1.43	0.16
	Restoration treatment: invader eradicated	1.01	0.76	1.33	0.19
	Restoration treatment: invaded	0.56	1.07	0.52	0.60
	Restoration treatment: never invaded	−0.26	0.71	−0.37	0.72

*Note*: Adjusted *R* ^2^ = −0.04.

### Multi‐decadal δ^15^N trends across invader‐eradicated islands using space‐for‐time substitution

Seabird guano δ^15^N values did not statistically differ between the islands where it was available (mean = 11.4‰ ± 1.6‰) (ANOVA: *F*_4,10_ = 2.38, *p* = 0.12), indicating that the baseline δ^15^N values entering the islands were comparable and island δ^15^N values could be compared. Again, Moutiti samples collected in 2022 were excluded from the analysis as they no longer provided a representative invaded island control with the colonization of seabirds.

**FIGURE 4:**
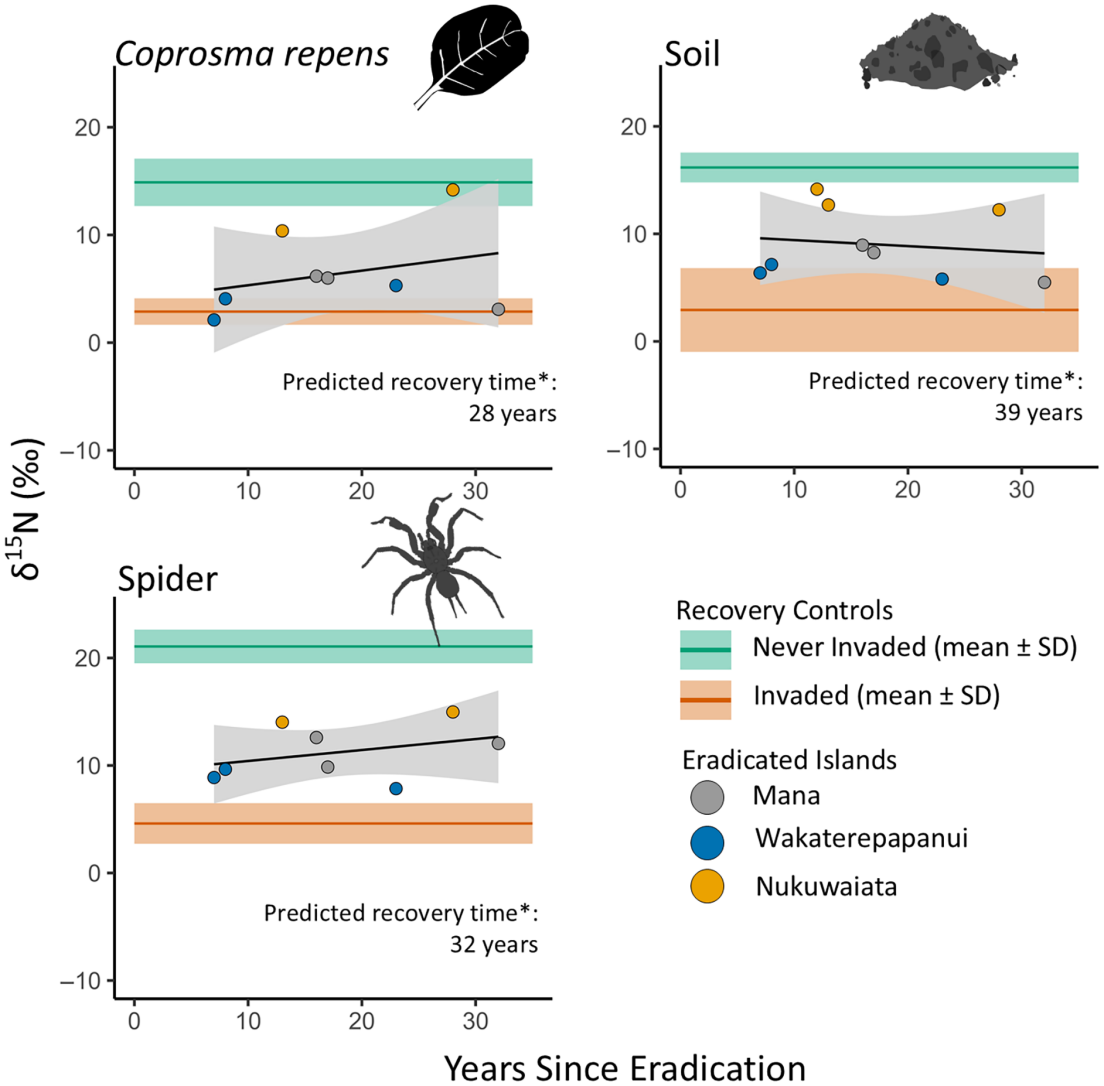
Nitrogen stable isotope (δ^15^N) values for exactly replicated *Coprosma repens*, soil, and spider samples collected 7–32 years following invasive rodent eradication (points) compared to mean ± 1 SD δ^15^N values for invaded (orange) and never‐invaded (green) islands. Invader‐eradicated island samples were collected outside seabird colonies to reflect whole‐island dynamics. Icon illustrations: P. Pascoe. *Predicted years to reach δ^15^N levels like a never‐invaded island from Jones (2010a).

The δ^15^N values on the invaded islands were significantly lower than those on never‐invaded islands (Figure 4), concurring with the findings of previous studies (Croll et al., 2005; Fukami et al., 2006; Jones, 2010a; Maron et al., 2006; Mulder et al., 2009). With invader‐eradicated and seabird‐restoration islands showing no difference in δ^15^N response, samples from Mana Island (invader eradicated in 1990) in addition to the two invader‐eradicated islands were used to look at δ^15^N trends over time since eradication for each sample type. There were no trends in δ^15^N with time since eradication for any of the sample types (Figure 4, Table 4). In general, δ^15^N values for samples from the invader‐eradicated islands were intermediate to those on never‐invaded and invaded islands, and some soil and *C. repens* samples were at similar δ^15^N levels to those found on never‐invaded islands (Figure 4, Table 4). Soil, *C. repens*, and spider δ^15^N levels on invader‐eradicated islands remain lower than those on never‐invaded islands (Figure 4), despite predictions that they would reach never‐invaded island levels 29 and 32 years post‐eradication for *C. repens* and spiders, respectively, on northern New Zealand islands (Jones, 2010a).

**TABLE 4:** Summary of linear model output for the relationship between the number of years since rodent eradication and δ^15^N values exactly replicated *Coprosma repens*, soil, and spider samples.

Response variable	Fixed effect	Estimate	SE	*T* value	*p* value	Adjusted *R* ^2^
*Coprosma repens* δ ^15^N	(Intercept)	5.84	2.37	2.46	0.03	−0.08
	Years since rodent eradication	0.00	0.11	−0.02	0.98	
Soil δ ^15^N	(Intercept)	10.76	1.81	5.93	<0.001	−0.004
	Years since rodent eradication	−0.09	0.09	−0.95	0.35	
Spider δ ^15^N	(Intercept)	9.59	1.88	5.11	<0.001	0.002
	Years since rodent eradication	0.10	0.10	1.02	0.32	

## DISCUSSION

To date, the study of island ecosystem change following invasive mammal eradications is uncommon, particularly over extended time periods. For the first time, we investigated long‐term changes in δ^15^N levels in island ecosystem components. We also investigated δ^15^N levels across, what is to the best of our knowledge, the longest temporal span of invader‐eradicated islands to date: 7–32 years post invasive rodent eradications. We found no significant changes in δ^15^N values across most islands irrespective of their restoration treatment, as well as no effect of sample type and seabird colony status on δ^15^N change. We also found no increase in δ^15^N in the 7‐ to 32‐year space‐for‐time substitution analysis; most ecosystem components on most invader‐eradicated islands did not reach levels of never‐invaded islands in the time frame predicted by previous work (see Jones, 2010a).

### Long‐term δ^15^N change on individual islands

The only island where an increase in δ^15^N was detected in the 16 years between the sampling events was Moutiti Island. When initially visited in 2006, there were no signs of seabird colonies on the island (Jones, 2010b). Despite no records of rodent control measures or seabird restoration activities occurring on the island in subsequent years, seabirds (kororā—blue penguins and potentially shearwaters) appear to have colonized the island at some point between then and the follow‐up visit in 2022. Breeding colonies of seabirds have previously been noted on rock stacks nearshore to Moutiti (Author's personal observation). Proximity to a source colony is a driver of recolonization (Buxton et al., 2014), so this could have enabled a colony to establish rapidly once conditions became favorable. Further surveys of the island are required to establish the nature and extent of the new colony and whether declines in invasive rodent numbers have made the island more suitable for seabird breeding. The significant increase in δ^15^N values across ecosystem components on this island would suggest that change in δ^15^N is able to capture dramatic changes in island ecosystems such as recolonization by seabirds.

No change in δ^15^N is the result that we expected for never‐invaded Takapourewa and Kuru Pongi, where seabird numbers are thought to be relatively stable (G. Taylor personal communication). These islands had the least variability in sample δ^15^N across the sampling events (SD ± 1.22%), further suggesting that ecosystem function on these islands has remained stable over the study period. The absence of δ^15^N change was unexpected across the invader‐eradicated and seabird‐restoration islands where we anticipated some increase over the study period. This lack of change may suggest that seabird numbers on these islands have not changed substantially over recent decades. Our colony density estimates from the restoration colonies on Mana and Te Hoiere were the highest of all the islands in 2022 (Table 1). However, as the restoration colonies still only take up a very small part of the islands, seabird densities may not yet have attained a level high enough to affect the whole island ecosystem (Jones, 2010b) as seabird‐driven nutrient effects can be very localized to a colony area (Pascoe et al., 2022).

Despite the invader‐eradicated and seabird‐restoration islands now being invasive mammal free, removing one of the largest threats faced by seabirds globally (Spatz, Holmes, et al., 2017), seabird populations may still fail to recover. Wakaterepapanui, which despite now being invasive rodent free since 1999, continues to have no detectable seabird colonies (Table 1), likely explaining the lack of δ^15^N change on this island. If the metapopulation of a species is in decline for other reasons, colony growth at a newly available site is likely to be minimal. The establishing colony will face similar extrinsic pressures to the broader population, and overall, there will be few prospecting birds to join the new colony (Buxton et al., 2014). Fairy prions, sooty shearwaters, and fluttering shearwaters are among the dominant seabird species across the study islands (Table 1). While the populations of each species are still large, sooty shearwaters are in rapid decline globally (BirdLife International, 2023), and the local New Zealand trends of each species are unknown.

### Long‐term δ^15^N trends across invader‐eradicated islands

In a separate study on islands spanning 12–22 years post rodent eradication in the Hauraki Gulf in northeastern New Zealand, Jones (2010a) predicted that it would take 28 years for *C. repens*, 32 years for spiders, and 39 years for soil samples to reach δ^15^N levels similar to those found on nearby, environmentally similar never‐invaded islands. With our 7‐ to 32‐year post‐eradication space‐for‐time substitution dataset, we were able to assess whether there was recovery of δ^15^N levels for soil, *C. repens*, and spiders in invader‐eradicated islands to pre‐invasion levels over multiple decades. While the islands in this study are in a different region of New Zealand from those studied by Jones (2010a), they are still temperate islands of similar sizes, and our previous work found no significant differences in isotope values between these islands and those in the Hauraki Gulf (Pascoe et al., 2021). However, the δ^15^N trends for the invader‐eradicated islands in this study did not match the predictions by Jones (2010a), with no significant increases in δ^15^N on islands with longer times since invasive mammal eradication. Nonetheless, we still found the invader‐eradicated islands in this study generally had δ^15^N values intermediate to those found on the invaded and never‐invaded islands (Figure 4), concurring with other studies from both temperate (Jones, 2010a, 2010b; Pascoe et al., 2021) and tropical (Benkwitt et al., 2021) island systems. This suggests that change following eradication has occurred, potentially within the first 7 years post‐eradication before sampling occurred, and that the recovery of ecosystem function on seabird islands may not be linear.

Following invasions, it has been hypothesized that some islands may be in an alternative stable state, where even after the eradication of the invasive species, hysteresis prevents the system from returning to its pre‐invasion state until a tipping point is reached (Mulder et al., 2009; Scheffer et al., 2001). A delay or absence in seabird population recovery and subsequent increase to sufficient densities to influence island‐wide nutrient dynamics could be the cause of this (Jones, 2010b). Seabird population growth is generally most rapid in the initial years following an eradication (de Brooke et al., 2017), and this initial spurt could lead to an initial rapid change in δ^15^N values. On islets of Palmyra Atoll in the Pacific Ocean, carbon and nitrogen isotopic shifts in crab diets were examined at two intervals after the eradication of black rats (*Rattus rattus*) (2 and 4 years) and were compared to those sampled 2 years before the eradication (Nigro et al., 2017). They found that the isotopic niche of the crabs expanded in this short post‐eradication interval. We detected an increase in δ^15^N values across all sample types on Moutiti Island, which went from no seabirds to a low‐density seabird colony between the two sampling periods. Contrastingly, Mana Island, which had established seabird colonies at the start of the study period from translocation programs of diving petrels, fairy prions, and fluttering shearwaters from 1993 to 2007/2008, underwent increases in all populations subject to translocation over the study period (Miskelly, 2023) but did not display any change in δ^15^N. This may indicate that while the initial recolonization of seabirds at a low density is reflected in δ^15^N change, a much greater change in seabird density is required to change δ^15^N to values typical of never‐invaded islands. This observation concurs with the findings of Jones (2010b) that high seabird densities are required to promote ecosystem function recovery. If this system exhibits alternative stable states, seabird density may need to reach thresholds higher than its original state for the system to shift back to that original state (Scheffer & Carpenter, 2003).

Seabird colonies also leave a nutrient legacy on islands with δ^15^N indicative of seabird nutrient subsidization present both outside the breeding season (Pascoe et al., 2022) and after islands have been abandoned (Kolb et al., 2010). This legacy could buffer the detection of population size changes on islands with a currently established seabird colony. Further studies investigating changes in seabird densities and δ^15^N values over time are required to better understand the linkage. Additionally, while we aimed to control for confounding variables influencing δ^15^N values by undertaking exact spatial and sample type replication between sampling years (Pascoe et al., 2022), it is likely that additional unconsidered variables, such as different levels of nutrient leaching or ammonia volatilization during each sampling event, may have also influenced the results.

## CONCLUSION

Globally, invasive mammal eradications are now common practice on islands, yet monitoring after eradication is not (Jones et al., 2016). Our results indicate that SIA_N_ may detect dramatic changes in seabird populations, such as the colonization of Moutiti Island by seabirds, but may not be able to detect ongoing population changes at relatively low numbers of seabirds. With samples collected from invader‐eradicated islands generally displaying δ^15^N values intermediate to those on invaded and never‐invaded islands, this result suggests that post‐eradication change has occurred, potentially rapidly within the first few years of eradication. Regular isotope sampling within the first 7 years of an eradication would shed light on this. A single measure of ecosystem recovery, be it population change in a taxon or a measure of ecosystem function, is insufficient to provide a complete view. Ideally, post‐eradication monitoring studies should couple isotope analysis with regular monitoring of seabird populations and other measures of ecosystem function to obtain a fuller understanding of ecosystem change and the influence of eradicating seabirds on other ecosystem components. Our findings highlight that previously predicted recoveries of isotope levels on invader‐eradicated islands to those similar to never‐invaded islands within three to four decades appear unrealistic in this system.

## AUTHOR CONTRIBUTIONS

*Conceptualization and design*: Penelope P. Pascoe, Justine Shaw, Rowan Trebilco, Christine K. Weldrick, and Holly P. Jones. *Data collection*: Mitchell Bartlett and Holly P. Jones. *Lab analysis*: Penelope P. Pascoe and Holly P. Jones. *Statistical analysis*: Penelope P. Pascoe. *Authored manuscript*: Penelope P. Pascoe. *Edits and comments on manuscript*: Penelope P. Pascoe, Justine Shaw, Rowan Trebilco, Christine K. Weldrick, Mitchell Bartlett, and Holly P. Jones.

## CONFLICT OF INTEREST STATEMENT

The authors declare no conflicts of interest.

## Supporting information


Appendix S1:

## Data Availability

Data and code (Pascoe & Jones, 2024) are publicly available in the University of Tasmania's Institute for Marine and Antarctic Studies (IMAS) Metadata Catalogue at https://doi.org/10.25959/VDN6-R115.
