# Supplementary material for: Decadal change in seabird‐driven isotopes on islands with differing invasion histories
Source: Ecol Appl. 2025 Jun 2;35(4):e70030. doi: 10.1002/eap.70030 (PMC12130745; doi:10.1002/eap.70030)
Supplement: Supplementary file 1 — Appendix S1: [file EAP-35-e70030-s001.pdf]

# Appendix S1

*Penelope P. Pascoe, Mitchell Bartlett, Justine Shaw, Rowan Trebilco, Christine K. Weldrick, and Holly P. Jones.* *Decadal change in seabird-driven isotopes on islands with differing invasion histories. Ecological Applications*

## Section S1. Model specifications

1. Effect of sampling event (2006/07 or 2002) on sample $\delta^{15}N$ values:

   sample $\delta^{15}N$ ~ sampling event x island + (1|sampling unit)

2. Effect of sample type, colony status and island restoration history on change in $\delta^{15}N$ for individual samples:

   New $\delta^{15}N$ - Original $\delta^{15}N$ values ~ sample type + colony status + restoration treatment

3. Effect of time since eradication on $\delta^{15}N$ for soil, spider and *Coprosma repens* samples across multiple eradicated islands:

   - soil $\delta^{15}N$ ~ years since eradication
   - spider $\delta^{15}N$ ~ years since eradication
   - *C. repens* $\delta^{15}N$ ~ years since eradication

## Table S1 - The $\delta^{15}N$ mean and standard deviation for each sample type collected on each island, inside or outside seabird colonies in the initial (2006/07) and new (2022) sampling events.

| Restoration Treatment | Island | Colony Status | Sample Type | No. Samples | New | | Initial | | Difference |
|---|---|---|---|---|---|---|---|---|---|
| | | | | | mean $\delta^{15}N$ | SD $\delta^{15}N$ | mean $\delta^{15}N$ | SD $\delta^{15}N$ | |
| Seabird Restoration | Mana | in | *C. repens* | 1 | 17.62 | | 17.43 | | 0.19 |
| Seabird Restoration | Mana | in | soil | 3 | 13.37 | 0.59 | 10.89 | 4.43 | 2.48 |
| Seabird Restoration | Mana | in | spider | 1 | 16.77 | | 19.50 | | -2.73 |
| Seabird Restoration | Mana | out | *C. repens* | 3 | 3.10 | 0.46 | 5.97 | 0.91 | -2.87 |
| Seabird Restoration | Mana | out | soil | 3 | 5.49 | 0.29 | 8.84 | 0.87 | -3.35 |
| Seabird Restoration | Mana | out | spider | 1 | 12.05 | | 10.77 | | 1.28 |
| Seabird Restoration | Maud | in | soil | 3 | 8.46 | 1.44 | 9.72 | 0.32 | -1.27 |
| Seabird Restoration | Maud | out | *C. robusta* | 2 | -0.06 | 3.39 | 2.45 | 0.96 | -2.51 |
| Seabird Restoration | Maud | out | soil | 3 | 4.68 | 0.58 | 4.50 | 3.27 | 0.18 |
| Seabird Restoration | Maud | out | spider | 1 | 8.03 | | 8.54 | | -0.51 |
| Eradicated | Nukuwaiata | in | *M. australis* | 2 | 11.68 | 3.19 | 10.21 | 4.91 | 1.47 |
| Eradicated | Nukuwaiata | in | soil | 2 | 11.88 | 0.35 | 12.01 | 0.52 | -0.13 |
| Eradicated | Nukuwaiata | out | *C. repens* | 1 | 14.19 | | 10.38 | | 3.81 |
| Eradicated | Nukuwaiata | out | *M. australis* | 3 | 8.98 | 2.69 | 11.04 | 3.38 | -2.06 |
| Eradicated | Nukuwaiata | out | soil | 3 | 12.24 | 2.61 | 13.43 | 2.91 | -1.19 |
| Eradicated | Nukuwaiata | out | spider | 1 | 14.98 | | 14.03 | | 0.95 |
| Eradicated | Wakaterepapanui | out | *C. repens* | 2 | 5.30 | 1.30 | 3.09 | 1.39 | 2.21 |
| Eradicated | Wakaterepapanui | out | soil | 3 | 5.80 | 1.64 | 6.29 | 1.95 | -0.49 |
| Eradicated | Wakaterepapanui | out | spider | 1 | 7.85 | | 9.46 | | -1.61 |
| Invaded | Moutiti | in* | *C. repens* | 3 | 13.37* | 0.96* | 4.66 | 0.40 | 8.71 |
| Invaded | Moutiti | in* | *M. australis* | 1 | 12.88* | | -1.80 | | 14.68 |
| Invaded | Moutiti | in* | soil | 3 | 15.97* | 0.98* | 13.03 | 6.25 | 2.94 |
| Invaded | Moutiti | out | *C. repens* | 3 | 12.89 | 1.96 | 2.88 | 1.23 | 10.00 |
| Invaded | Moutiti | out | soil | 2 | 17.32 | 0.26 | 8.72 | 1.77 | 8.60 |
| Invaded | Moutiti | out | spider | 1 | 19.99 | | 6.31 | | 13.69 |
| Invaded | Tawhitinui | out | *M. australis* | 3 | -6.24 | 3.91 | -6.07 | 1.04 | -0.17 |
| Invaded | Tawhitinui | out | soil | 3 | 0.47 | 1.49 | 2.22 | 3.39 | -1.75 |
| Invaded | Tawhitinui | out | spider | 1 | 4.26 | | 3.82 | | 0.44 |
| Never Invaded | Kuru Pongi | in | *C. repens* | 3 | 15.39 | 2.64 | 16.24 | 2.50 | -0.85 |
| Never Invaded | Kuru Pongi | in | soil | 3 | 15.41 | 0.79 | 16.51 | 1.22 | -1.10 |
| Never Invaded | Kuru Pongi | in | spider | 1 | 21.45 | | 22.21 | | -0.76 |
| Never Invaded | Kuru Pongi | out | *C. repens* | 3 | 12.72 | 0.55 | 14.47 | 0.77 | -1.76 |
| Never Invaded | Kuru Pongi | out | soil | 3 | 15.89 | 0.87 | 16.43 | 0.74 | -0.53 |
| Never Invaded | Takapourewa | in | *M. australis* | 2 | 13.55 | 1.36 | 13.61 | 0.82 | -0.06 |
| Never Invaded | Takapourewa | in | soil | 2 | 15.91 | 1.36 | 17.80 | 1.57 | -1.90 |
| Never Invaded | Takapourewa | in | spider | 1 | 20.67 | | 21.73 | | -1.06 |
| Never Invaded | Takapourewa | out | soil | 2 | 14.90 | 3.55 | 15.08 | 0.04 | -0.18 |

* These sampling locations on Moutiti/Victory were in newly established seabird colonies in 2022, but there was no evidence of colonies at these same locations at the initial sampling even in 2007
